# Tracheal ring fracture and early tracheomalacia following percutaneous dilatational tracheostomy

**DOI:** 10.1186/1472-6815-5-6

**Published:** 2005-08-31

**Authors:** Eu Chin Ho, Atul Kapila, William Colquhoun-Flannery

**Affiliations:** 1 Department of Otolaryngology, Royal Berkshire Hospital, Reading, Berkshire, RG1 5AN, UK; 2 Department of Anaesthetics, Royal Berkshire Hospital, Reading, Berkshire, RG1 5AN, UK

## Abstract

**Background:**

Percutaneous dilatational tracheostomy (PDT) is increasingly popular within intensive care units for patients who need prolonged ventilatory support. Significant complications are rare.

**Case presentation:**

Our patient suffered tracheal ring fracture and early tracheomalacia following this procedure. These complications are demonstrated in our accompanying video.

**Conclusion:**

Contrary to common beliefs, tracheal rings are commonly fractured during the PDT procedure. The consequent granulation can lead to tracheal stenosis and tracheomalacia.

## Background

In many intensive care units within the United Kingdom, the Percutaneous Dilatational Tracheostomy (PDT) technique had replaced the traditional open (surgical) tracheostomy method for patients who need prolonged ventilatory support. This technique is often performed by the intensivists themselves, hence is time and cost effective. 2 complications of this technique are described and shown in a video accompanying this report.

## Case presentation

Our patient was a 78 year old lady who following a total hip replacement developed a pneumothorax and pneumonia and needed post-operative ventilation. She was difficult to wean off the ventilator and on day 7 post-op, we decided to perform a combined open and percutaneous dilatational tracheostomy (PDT). Due to the difficult anatomy, we dissected down to the level of the strep muscles before proceeding with PDT technique. The tracheal was easily palpated by this stage. We used a Portex ULTRAperc (Portex Ltd, Hythe, Kent, England) kit, with a size 8.0 mm ID tracheostomy tube. Her tracheal rings were rather calcified and we heard a 'crack' during the dilatation process. Nevertheless, there was no problem with introducing the tracheal tube. The procedure was carried out under guiding vision from within the trachea by flexible video bronchoscopy with no obvious complications noted.

**Figure 1:**
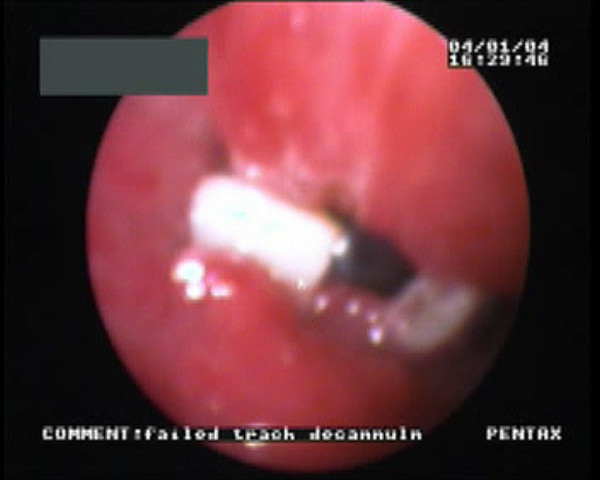
Fractured tracheal ring prolapsing into tracheal lumen above tracheostomy tube.

Subsequently this lady failed three attempts at tracheal decannulation. She would develop immediate airway obstruction following tracheostomy tube removal requiring reinsertion of the tracheostomy tube. On day 32 of her ICU stay, following the third attempt, we performed a video endoscopic airway examination. Passing the flexible bronchoscope (Pentax FB-18BS, Pentax Medical, Slough, England) through the larynx, we found a fractured tracheal cartilage ring, protruding into the trachea just above the tracheostomy tube. Then, by passing the flexible bronchoscope through the tracheostomy tube, we saw tracheal wall oedema and inflammation. There was also collapse of the anterior tracheal wall on inspiration demonstrating tracheomalacia. This segment of tracheal wall collapse was quite long as shown by the partial removal of the tracheostomy tube.. [Figure 1 & see video file 1]

She was eventually discharged to a ward environment on day 38 with the tracheostomy tube in situ. Unfortunately she later suffered a cardio-respiratory arrest and the patient died. The post mortem findings were consistent with that of respiratory failure secondary to bronchopneumonia.

## Discussion

Our patient had a tracheal ring fracture and early distal tracheomalacia following a PDT procedure. It is our opinion that the tracheal ring fracture would have been caused during the PDT procedure, either by the dilator or by the tracheostomy tube itself. Why did the bronchoscopist not spot this at the time of the procedure? As the fractured tracheal ring was sitting above the tracheostomy tube, it was possible that the in-situ translaryngeal endotracheal tube at the time of the procedure could have prevented displacement of the fractured fragments. Even though video bronchoscopy guidance is virtually routine nowadays, perhaps it is not 100% failsafe.

It is often believed that tracheal rings are displaced but remain intact with the PDT technique. In one study, PDT was performed on cadaveric specimens, which demonstrated significant peristomal mucosal tear and cartilaginous fracture with this technique [1]. Well calcified tracheal rings would have made this complication more likely. The consequent granulation and scarring can lead to tracheal stenosis at the subglottic, suprastomal or stomal level [1,2]. Stenosis at a suprastomal level is believed to be caused by anterior tracheal wall injury and granulation formation from the tracheostomy procedure and tube itself [2].

Autopsies of patients who had tracheostomy also showed necrosis of the tracheal cartilage and mucosal ulceration beyond the tracheostomy site as early as 2 weeks post cannulation [3]. Together with the irritating effects of the tracheostomy cuff, these changes are likely to be responsible for the distal tracheomalacia seen in our patient. Other possible causes of tracheomalacia would include the use of oversized cannula and infection. However, it is important to keep things in perspective, as these complications are rare.

We tried to obtain consent for publishing this case report from the patient's next of kin. However, all attempts to contact the patient's family were unsuccessful.

## Conclusion

Contrary to common beliefs, tracheal rings are commonly fractured during the PDT procedure. The consequent granulation can lead to tracheal stenosis and tracheomalacia. Nevertheless, by understanding the pathophysiology behind the complications, these risks could be minimised.

## Competing interests

The author(s) declare that they have no competing interests.

## Authors' contributions

**ECH **was overall coordinator and drafted, revised and background researched the paper.

**AK **made the video recording of the complications and critically revised the article.

**WC **supervised the project and critically revised the article.

## Pre-publication history

The pre-publication history for this paper can be accessed here:



## Supplementary Material

Additional File 1 Video demonstrating the 2 complications in this patient. Firstly, the video demonstrated the fractured tracheal ring prolapsing into tracheal lumen. This was recorded by passing a fiberoptic bronchoscope per nasally. Later in the video, distal tracheomalacia was demonstrated by passing the fiberoptic bronchoscope through and partially withdrawing the tracheostomy tube. Click here for file
